# Epidemiology of Sports-Related Eye Injuries Among Athletes in Tianjin, China

**DOI:** 10.3389/fmed.2021.690528

**Published:** 2021-09-16

**Authors:** Jingkai Zhang, Xinlei Zhu, Zhiyong Sun, Jiaxing Wang, Zhuoyu Sun, Jianan Li, Yunli Huang, Tian Wang, Ruotian Xie, Han Han, Xiao Zhao, Yinting Song, Miao Guo, Tiantian Yang, Haokun Zhang, Kai He, Yiming Li, Yi Lei, Yanfang Zhu, Bohao Cui, Yuyang Miao, Bo Huang, Rodrigo Brant, Hua Yan

**Affiliations:** ^1^Department of Ophthalmology, Tianjin Medical University General Hospital, Tianjin, China; ^2^Shandong Eye Institute, Shandong First Medical University and Shandong Academy of Medical Sciences, Qingdao, China; ^3^Department of Ophthalmology, Emory University, Atlanta, GA, United States; ^4^Department of Epidemiology and Statistics, School of Public Health, Tianjin Medical University, Tianjin, China; ^5^Department of Ophthalmology, University of Mississippi Medical Center, Jackson, MS, United States; ^6^Department of Ophthalmology and Visual Sciences, Federal University of São Paulo, São Paulo, Brazil

**Keywords:** eye injury, athletes, age, family income, training

## Abstract

**Purpose:** To investigate the incidence, characteristics, and risk factors of sports-related eye injuries among athletes in Tianjin, China.

**Methods:** A cross-sectional study was carried out from March 2018 to October 2018. In this study, the athletes from Tianjin University of Sports, Tianjin Vocational College of Sports, and Tianjin provincial sports teams were selected for general investigation. In total, 1,673 athletes were invited and 1,413 participated in the study (response rate of 84.5%).

**Results:** In total, 1,413 athletes were enrolled; 151 had suffered from sports-related eye injuries, with an incidence of 10.7% (95% *CI*: 9.1–12.0%). Handball (38.5%) was the sport with the highest incidence of eye injuries, followed by water polo (36.4%) and diving (26.7%). Overall, 42.4% of the athletes were injured by ball and 22.5% of injuries came from teammates. The eye injuries usually occurred during training (64.2%) and competitions (14.6%). Adnexa wound (51.7%) was the most common type of injury. About 11.9% of the athletes with eye injuries had the impaired vision; 66.7% failed to see doctors on time. The athletes <18 years of age had a higher risk of eye injuries (odds ratio [*OR*] =1.60, 95% *CI*: 1.06–2.40). The athletes with lower family income (<1,000 RMB) were at risk population for sports-related eye injuries (*OR* = 3.91, 95% *CI*: 2.24–6.82). Training >4 h a day increased the risk of eye injuries (*OR* = 2.21, 95% *CI*: 1.42–3.43).

**Conclusion:** The incidence of sports-related eye injuries among athletes was 10.7% in Tianjin, China. Handball, water polo, and diving were the most common activities of injury. Age, family income, and training time were the risk factors for sports-related eye injuries.

## Introduction

Sports-related eye injuries were important eye conditions encountered by ophthalmologists since 8.3–17% of eye injuries were sports-related. These eye injuries might lead to blindness because sports accounted for one-third of blindness cases due to ocular trauma (1–4), especially among adolescents (5–8). Therefore, sports-related eye injuries represented a huge burden on the individuals, families, and healthcare systems (9). Sports-related eye injury was of concern considering the emphasis placed on the importance of practicing sports to prevent chronic diseases. Although about 90% of sports-related eye injuries were preventable with adequate protective devices (10). Most people did not have the awareness of the importance of wearing adequate eye protection and the eye injuries should be evaluated on-site with an adequate examination of the eye and adnexa (11, 12). About 42% of people who had ever participated in sports have suffered eye injuries (13), but only 9.4% of those patients were using protective devices during their activities (2).

In the joint policy statement issued by the American Academy of Ophthalmology and the American Academy of Pediatrics in 2004, the eye-injury risk of a sport to the unprotected player was roughly categorized as high risk, moderate risk, low risk, and eye safe (14). However, because of the differences in the types and frequency of sports among countries, sports-related eye injuries had certain geographical and cultural characteristics. The most common causes of eye injuries varied from basketball in America (1), baseball in Korea (2), floorball in Finland (3), and soccer in Israel (15). Nevertheless, no epidemiological data were available from China, which was the most populous country in the world. With the economic development of the Chinese society, more people took part in sports to keep healthy (16), but emergency rooms in China were facing a rise in the numbers of patients with sports-related injuries than before (17, 18). This new state prompted the need to investigate the current situation and risk factors of sports-related eye injuries in China and proposed preventive strategies.

As a high-risk population of sports-related eye injuries, athletes should receive more attention (19). Eye injuries happening to the athletes might be more serious and complex because of the high level of the sports and might have severe repercussions on their entire professional career. To our knowledge, no previous studies specially examined the eye injuries of athletes in China. Therefore, the aim of this study was to investigate the epidemiology and characteristics of sports-related eye injuries among athletes in Tianjin, China, and to explore the risk factors associated with those injuries. The results will benefit the athletes in preventing eye injuries during their activities and will be helpful in convincing the stakeholders of the importance of eye injury in athletic-level sports.

## Methods

### Participants

This cross-sectional study was conducted from March 2018 to October 2018. The athletes from Tianjin University of Sport, Tianjin Vocational College of Sports, and Tianjin provincial sports teams were chosen for general investigation. Twenty-five sports were investigated, including soccer, basketball, badminton, baseball, track and field, swimming, weightlifting, volleyball, taekwondo, judo, tennis, table tennis, wrestling, Ryukyu, water polo, diving, synchronized swimming, martial arts, cycling, art gymnastics, handball, field hockey, archery, shooting, and fencing. The athletes with <1 year of training were excluded from the study. The study was performed according to the Declaration of Helsinki and was approved by the ethics committee of Tianjin Medical University General Hospital. All participants signed the informed consent form.

### Data Collection

The athletes were asked to complete a questionnaire about socio-demographic characteristics (sex, age, height, weight, myopia, education level, monthly family income, and sleep deficiency), sports-related information (training activities, training age, daily training time, and protective device use), and history of ocular trauma. Body mass index (BMI) was calculated as weight (kg) divided by height squared (m^2^), and was categorized as underweight (<18.5 kg/m^2^), normal weight (18.5–23.9 kg/m^2^), and overweight/obesity (≥24.0 kg/m^2^). Sleep deficiency was defined as <8 h of sleep daily.

The detailed data about the history of ocular trauma were recorded by ophthalmologists. Sports-related eye injuries were defined as ocular trauma that occurred during the training sessions or competitive events over the past year. The athletes with ocular trauma unrelated to sports were excluded from the study. The collected data included laterality, accident location, cause of injury, type of injury, visual acuity after injury, time for medical help, and treatment, which were obtained by consulting the medical records of the sports team and confirming to the participants. The participants received comprehensive ophthalmic examinations, which included best corrected visual acuity (BCVA), refraction, intraocular pressure, slit lamp biomicroscopy examination, and fundus examination.

### Statistical Analysis

The incidence of eye injuries was calculated by injuries/total in different sports. The athletes with and without the sports-related eye injuries were compared with respect to socio-demographic characteristics, myopia, training age and time, sleep deficiency, and protective device use, using the chi-square test. Athletes with sports-related eye injuries were classified as the younger group (<18 years) and the older group (≥18 years). Laterality, cause, accident location, and type of sports-related eye injuries were compared between the younger and older groups. Using multivariable logistic regression analysis, the odds ratios (*OR*s) and 95% *CI*s of sports-related eye injuries in relation to sex, age, BMI, education level, family income, training age and time, myopia, and protective devices use were determined. The differences were considered statistically significant when *P* ≤ 0.05. All the analyses were carried out using SPSS 20.0 (IBM, Armonk, NY, USA).

## Results

### Characteristics of the Participants

**Figure 1:**
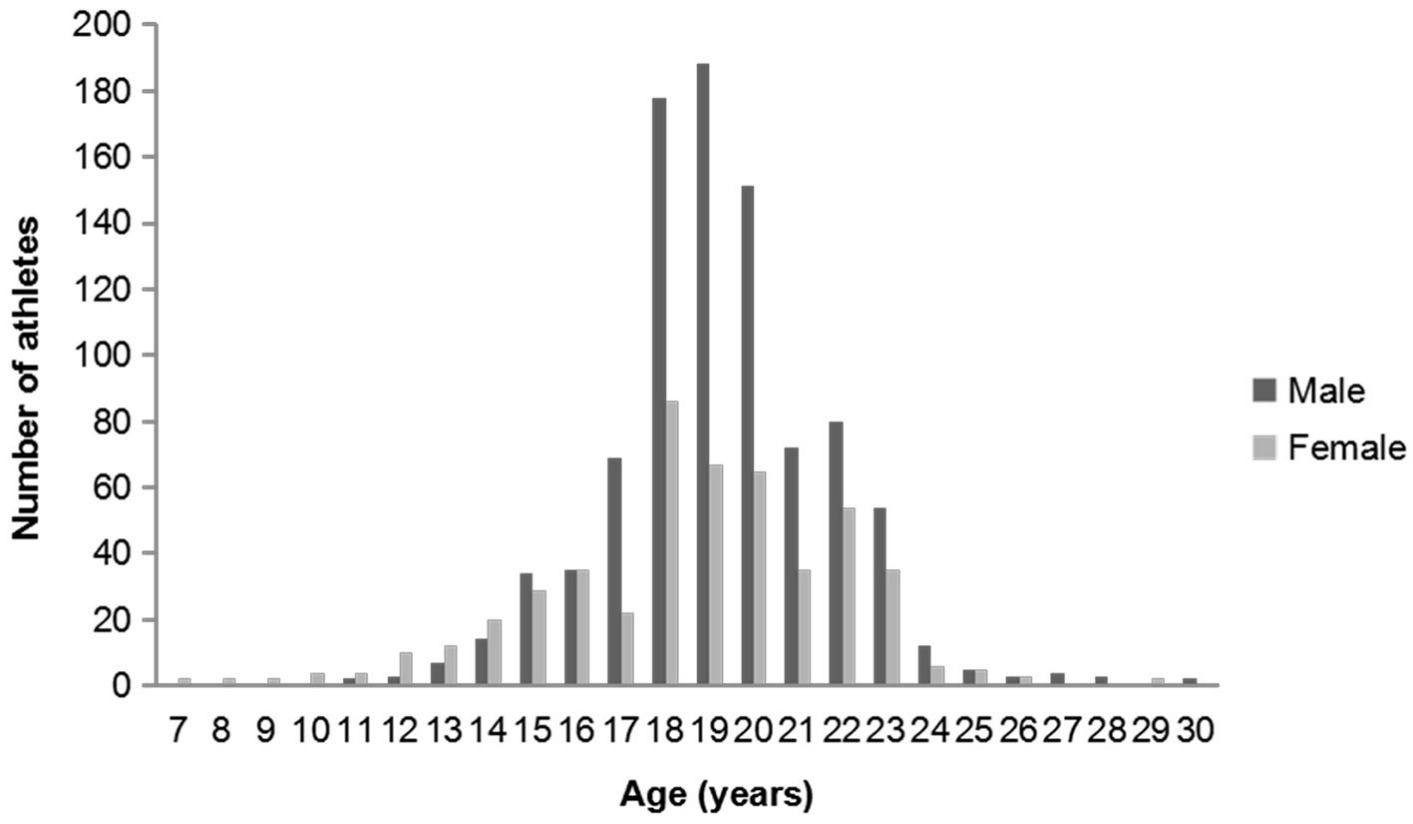
Age distribution of all the athletes by sex.

In this study, we intended to investigate all 1,673 athletes registered in Tianjin University of Sport, Tianjin Vocational College of Sports, and Tianjin provincial sports teams. However, since some athletes were not in Tianjin for various reasons during the investigation, only 1,433 athletes were invited to participate in the study, and 1,413 athletes returned the questionnaires and completed the eye examinations, for a total response rate of 84.5%. Among the 1,413 athletes, 914 (64.7%) were male and 499 (35.3%) were female. The mean age of the participants was 19.0 ± 2.8 (range, 7–30) years (Figure 1). The average age, height, and weight were 19.3 ± 2.4 years, 179.6 ± 7.7 cm, 72.2 ± 12.7 kg in male and 18.6 ± 3.3 years, 167.4 ± 9.0 cm, 59.6 ± 11.9 kg in female, respectively.

### Sports-Related Eye Injuries

**Figure 2:**
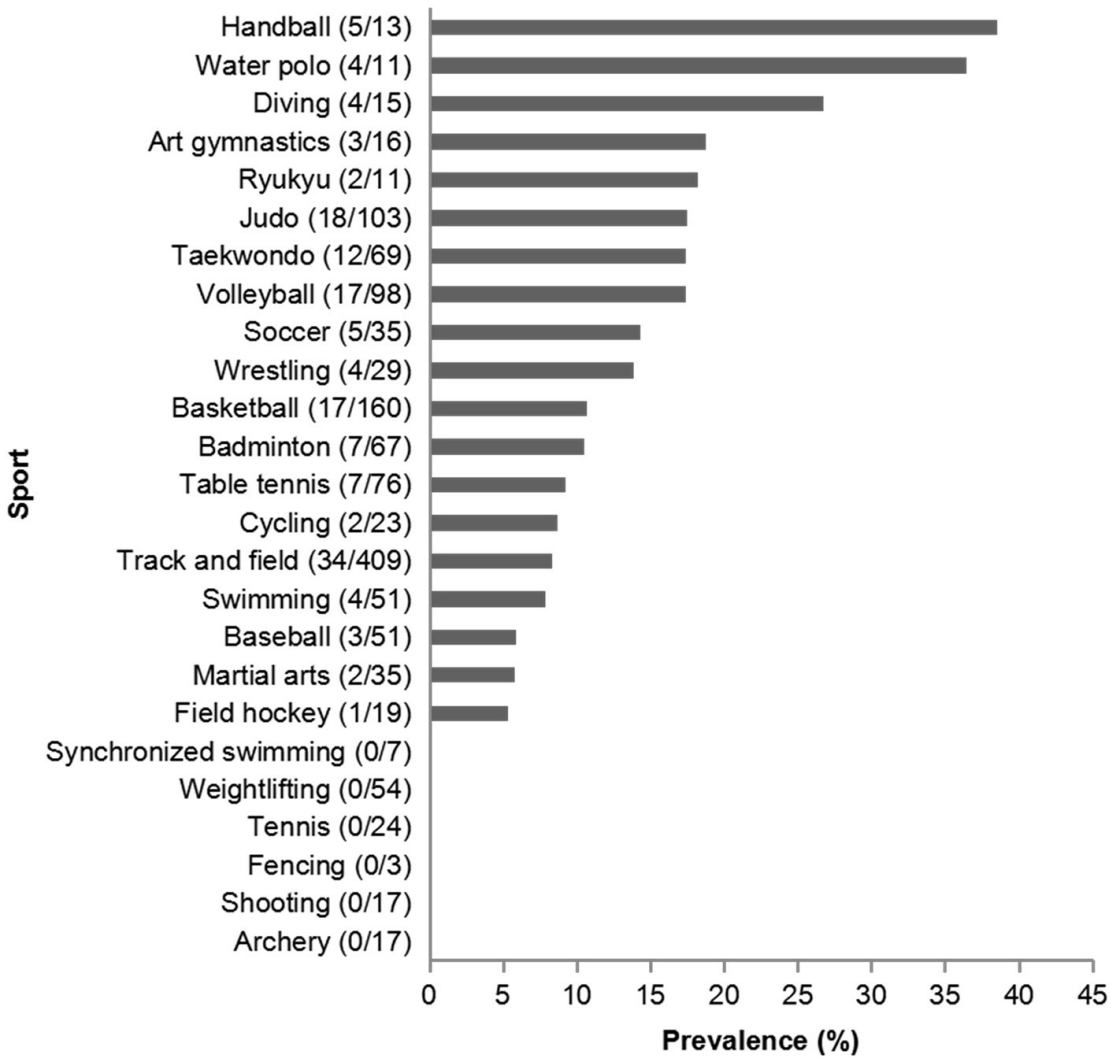
The incidence of eye injuries in different sports. The number in the parentheses was the athletes (injury/total) for each sport.

In total, 151 athletes reported sports-related eye injuries in this study, for the incidence of 10.7% (95% *CI*: 9.1–12.0%). Among 914 male athletes, 100 athletes with sports-related eye injuries were reported, the incidence was 10.9%, and the proportion in female was 10.2% (51 injuries in 499 athletes). Handball (38.5%) was the sport with the highest incidence of eye injuries, followed by water polo (36.4%), and diving (26.7%) (Figure 2). The incidence was significantly different among the sports (*P* < 0.001).

The athletes with sports-related eye injuries were younger, had lower education levels, had lower monthly family income, and had longer daily training time than those without sports-related eye injuries (Table 1). No significant differences were found in sex, BMI, myopia, training age, sleep deficiency, and protective device use.

**Table 1:** Characteristics of athletes with and without sports-related eye injuries.

**Variable**	**Sports-related eye injury**		***P***
	**Yes (** ***n*** **= 151)**	**No (** ***n*** **= 1,262)**	
**Age (years)**, ***n*****(%)**			< 0.001
<18	54 (35.8)	250 (19.8)	
<18	97 (64.2)	1,012 (80.2)	
**Sex**, ***n*****(%)**			0.675
Female	51 (33.8)	448 (35.5)	
Male	100 (66.2)	814 (64.5)	
**Body mass index (kg/m^2^)**, ***n*****(%)**			0.166
<18.5	15 (9.9)	129 (10.2)	
18.5–23.9	95 (62.9)	874 (69.3)	
≥24.0	41 (27.2)	259 (20.5)	
Myopia, *n* (%)	123 (81.5)	1,052 (83.4)	0.555
**Education level**, ***n*****(%)**			0.001
Primary school and below	3 (2.0)	25 (2.0)	
Junior high school	21 (13.9)	92 (7.3)	
Senior high school	36 (23.8)	195 (15.5)	
University and above	91 (60.3)	950 (75.3)	
**Monthly family income**^a^**(RMB)**, ***n*****(%)**			< 0.001
<1,000	26 (17.2)	96 (7.6)	
1,000–2,000	22 (14.6)	189 (15.0)	
2,001–5,000	63 (41.7)	490 (38.8)	
>5,000	40 (26.5)	487 (38.6)	
**Training age (years)**, ***n*****(%)**			0.364
<5	54 (35.8)	521 (41.3)	
5–10	80 (53.0)	627 (49.7)	
>10	17 (11.3)	114 (9.0)	
**Daily training time (h)**, ***n*****(%)**			< 0.001
<2	42 (27.8)	505 (40.0)	
2–4	22 (14.6)	330 (26.1)	
>4	87 (57.6)	427 (33.8)	
Sleep deficiency, *n* (%)	64 (42.4)	527 (41.8)	0.883
Use of protective devices, *n* (%)	19 (12.6)	153 (12.1)	0.871

a *Family income: average monthly income per person*.

In athletes with sports-related eye injuries, 80.1% of the injuries were monocular and 19.9% were binocular. Overall, most injuries were caused by the ball, which accounted for 42.4%, while 22.5% of eye injuries were caused by the teammates. Violent collisions and tumble, each accounted for 10.6% of injuries. In general, 64.2% of the eye injuries occurred during training, and 14.6% happened in the competition. Adnexa wound (51.7%) was the most common type of injury, followed by contusion (29.1%) and corneal abrasion (17.9%) (Table 2). The injuries, such as penetrating injuries, globe rupture, perforating injuries, intraocular foreign body injuries, and lamellar laceration were not found in the athletes with eye injuries in the present study and were therefore not listed in Table 2.

**Table 2:** Causes, locations, and types of sports-related eye injuries in athletes, stratified by age and sex.

**Variable**	**Athletes with sports-related eye injury**					
	**Total (*n* = 151)**	** <18 years (*n* = 54)**	**>18 years (*n* = 97)**	**Male (*n* = 100)**	**Female (*n* = 51)**	***P***
**Laterality**, ***n*****(%)**						0.246
Monocular injury	121 (80.1)	46 (85.2)	75 (77.3)	81 (81.0)	40 (78.4)	
Binocular injury	30 (19.9)	8 (14.8)	22 (22.7)	19 (19.0)	11 (21.6)	
**Cause of injury**, ***n*****(%)**						< 0.001
Ball	64 (42.4)	18 (33.3)	46 (47.4)	43 (43.0)	21 (41.2)	
Teammate	34 (22.5)	12 (22.2)	22 (22.7)	21 (21.0)	13 (25.5)	
Violent collision	16 (10.6)	6 (11.1)	10 (10.3)	14 (14.0)	2 (3.9)	
Tumble	16 (10.6)	8 (14.8)	8 (8.2)	13 (13.0)	3 (5.9)	
Breach of rules	11 (7.3)	0 (0)	11 (11.3)	7 (7.0)	4 (7.8)	
Other causes	10 (6.6)	10 (18.5)	0 (0)	2 (2.0)	8 (15.7)	
**Accident location of injury**, ***n*****(%)**						0.174
Training ground	97 (64.2)	38 (70.4)	59 (60.8)	59 (59.0)	38 (74.5)	
Competition field	22 (14.6)	4 (7.4)	18 (18.6)	13 (13.0)	9 (17.6)	
Other places	32 (21.2)	12 (22.2)	20 (20.6)	28 (28.0)	4 (7.8)	
**Type of injury**, ***n*****(%)**						0.189
Adnexa wound	78 (51.7)	34 (63.0)	44 (45.4)	49 (49.0)	29 (56.9)	
Contusion	44 (29.1)	12 (22.2)	32 (33.0)	29 (29.0)	15 (29.4)	
Corneal abrasion	27 (17.9)	7 (13.0)	20 (20.6)	20 (20.0)	7 (13.7)	
Orbital fracture	2 (1.3)	1 (1.9)	1 (1.0)	2 (2.0)	0 (0)	

After stratification for age, younger women and older men more often suffered from eye injuries. The mean age of athletes with eye injuries was 19.2 ± 2.8 years for men and 17.4 ± 4.3 years for women. In the younger group, except for ball and teammate as the most common causes of injuries, the tumble and other causes were reported as the third more frequent reasons for eye injuries. Breach of rules was the third common cause in the older groups (Table 2).

Among the athletes engaged in track and field activities, adnexa wounds accounted for 54.2% of injuries. Contusion (41.2%) was the most common type of injury in basketball. About 11.9% of the athletes with eye injuries had impaired vision, and 66.7% failed to see doctors in time. In total, 22 of 151 (14.6%) athletes were operated in a consequence of the eye injury.

### Risk Factors for Sports-Related Eye Injury

The risk factors of sports-related eye injuries were listed in Table 3. The multivariable logistic regression analysis showed that athletes <18 years of age (*OR*: 1.60, 95% *CI*: 1.06–2.40), athletes with lower monthly family income (<1,000 RMB) (*OR*: 3.91, 95% *CI*: 2.24–6.82), and athletes training >4 h a day (*OR*: 2.21, 95% *CI*: 1.42–3.43) had a higher risk of suffering from eye injuries. On the other hand, sports-related eye injuries were not associated with sex, myopia, education level, BMI, training age, sleep deficiency, and use of protective devices.

**Table 3:** Factors statistically associated with sports-related eye injuries in athletes.

**Variable**	**Multivariable logistic regression**	
	**Crude OR (95%CI)**	**Adjusted OR (95%CI)^b^**
Age (years)		
>18	1.00	1.00
<18	2.25 (1.57–3.23)	1.60 (1.06–2.40)
Monthly family income*^a^* (RMB)		
>5,000	1.00	1.00
2,001–5,000	1.57 (1.03–2.37)	1.51 (0.99–2.30)
1,000–2,000	1.42 (0.82–2.45)	1.73 (0.99–3.03)
<,1000	3.30 (1.92–5.66)	3.91 (2.24–6.82)
Daily training time (h)		
<2	1.00	1.00
2–4	0.80 (0.47–1.37)	0.75 (0.44–1.30)
>4	2.45 (1.66–3.62)	2.21 (1.42–3.43)

a *Family income: average monthly income per person*.

b *Adjusted for age, sex, body mass index, myopia, education level, monthly family income, training age, daily training time, sleep deficiency, and use of protective devices*.

*Only variables with statistical significance were shown*.

## Discussion

To the best of our knowledge, this is the first study to investigate the epidemiology, characteristics, and risk factors of sports-related eye injuries among athletes in Tianjin, China. In the present study, the incidence of sports-related eye injuries was 10.7% (95% *CI*: 9.1–12.0%) in athletes, and about 11.9% of them had impaired vision. The eye injuries occurred most frequently when playing handball, playing water polo, and diving. Around 65% of the eye injuries were caused by the ball and teammates. The majority of eye injuries (64.2%) occurred during training. The most common types of eye injuries were adnexa wounds, contusion, and corneal abrasion. Younger age, lower family income (<1,000 RMB), and longer training time (>4 h/day) were associated with a higher risk of sports-related eye injuries in the athletes.

The studies had pointed out that sports-related eye injuries were preventable when using adequate protective devices and adopting appropriate comportment/attitude (20). However, ocular trauma was commonly seen in the emergency rooms and might have serious consequences on the life and professional career of the athletes (12, 21, 22). The patients with sports-related eye injuries visited emergency departments every 13 min in the United States (23). About 11% of the eye injuries led to the long-term functional visual impairment (3) and 28% of the cases of post-traumatic eyeball enucleation were related to sports (24, 25).

In the present study, 151 out of 1,413 athletes reported sports-related eye injuries, resulting in an incidence of 10.7% (95% *CI*: 9.1–12.0%). Around 12% of those with sports-related ocular trauma had impaired vision, but 66.7% of them failed to seek eye healthcare in time. It was reported that only 3.1% of patients with eye injuries have impaired vision in the emergency departments (1), which was lower than in the present study. In addition, the present study was among the rare studies to report the visual impairment of athletes with an eye injury. Our sports teams had complete healthcare record information that recorded the annual physical examination of athletes, including ocular examination. It was not only convenient for us to understand the past ocular conditions of athletes and increase the reliability of the research data but also conducive for coaches to pay close attention to the physical changes of the athletes, which was worthy of extensive learning and reference.

Among the available studies, the sport causing eye injury varied among countries, probably because of the varying popularity of the sports across the world. Moon et al. (2) reported that baseball was the most common sport for eye injuries in Korea, followed by football, hiking, and badminton. In Finland, floorball, soccer, and tennis were the major sports causing eye injuries (3). In the United States, basketball was the main sport leading to eye injury (1). The present study was the first to report epidemiological data about sports-related eye injury in Tianjin, China, and showed that the injuries occurred most commonly as a result of playing handball, followed by water polo and diving. The athletes playing water polo lacked good sight because of the water and goggles and were at higher risk of ball impact and body collision, which resulted in more injuries than during other activities. The incidence of eye injuries in art gymnastics was 18.8%, which was beyond our expectations. We found that eye injuries in art gymnastics mostly came from accidental eyelid poking by ribbons.

Myopia was an important eye problem in China, with an alarming increase in the prevalence of high and very high myopia among teenagers in the last 15 years (26). The prevalence of myopia was 83.2%, which was similar to the previous studies (27, 28). Although myopia was not related to eye injuries, it should draw enough attention because of such a high prevalence and the need to wear some kind of corrective lenses when practicing sports or taking the risk of not seeing the play and the potential threats. It was worthy of our attention that 46.8% of the athletes in this study did not realize their myopia status, which might further increase the potential risks for eye injuries. These results suggested that the athletes should be screened for myopia before starting their careers.

Multiple causes of injury could be found in sports-related eye injuries. Among high school and collegiate athletes, it was reported that most of the eye injuries were caused by contact; in particular, player contact was the leading cause of eye injuries, accounting for 47.3% in high school and 64.5% in college, while the proportion of ball contact was 37.1% in high school and 18.3% in college (19). In the present study, ball contact (42.4%) and teammates (22.5%) were also the most common causes of eye injuries. Among the athletes <18 years of age, 33.3% of eye injuries were caused by ball contact and 22.2% were caused by teammates. Among the older athletes, most of the injuries were also caused by ball and teammates, but the frequency of ball contact was higher (47.4%), while that of teammates was similar (22.7%). Using eye protective equipment would effectively reduce the damage caused by the ball. Boden et al. (19) reported that eye injuries caused by ball accounted for 72.7% in the field of hockey, but after forcing the players to wear eye-protective devices, the proportion dropped by 52%.

The types of eye injuries varied with the causes of injuries. Haring et al. (1) found that adnexa wound and contusion were the most common types of injuries after analyzing data from the Nationwide Emergency Department Sample (NEDS) in the United States, which was similar to our results. Boden et al. (19) also reported that contusion accounted for the largest proportion of eye injuries among the athletes in high school and college and that those contusions were closely related to ball and player contact.

According to the multivariable analysis, athletes <18 years of age had a higher risk of eye injury than those >18 years. Younger athletes might lack the awareness for eye protection and related knowledge during training, and might be brasher in the practice of their sport. Elevated risk was also found in athletes with lower family income (<1,000 RMB). With per capita disposable income reaching 28,228 RMB in China in 2018, such a low family income might affect athletes in many aspects, such as sports equipment, nutrition, health condition, and living environment, which might greatly affect athletic performance during training. On the other hand, the health behavior of athletes was affected by socio-economic status leading that lower-income athletes might not pay attention to eye protection. Training time was also a risk factor for eye injuries. Of course, more training increases the likelihood of injury, and athletes training >4 h daily showed a substantially elevated risk of eye injury. Long-time training every day might cause fatigue and tiredness, which could increase the risks of ocular injury. Therefore, it was necessary to improve the awareness of eye protection during activities, especially for younger athletes and those in lower socio-economic status (1). Besides, providing eye protection devices for those who had longer training time might be an effective measure. Specific interventions should be designed and tried in the future.

Many, although not all, sports-related eye injuries were preventable with adequate protective equipment, which had been reported in the previous studies (10, 20). Nevertheless, only 172 of the 1,413 athletes included in the present study were using protective devices during their activities, which accounted for 12.2% only. Since the athletes were mostly engaged in swimming, shooting, fencing, and cycling, regrettably, we could not draw the above conclusion under the overall analysis. In athletes with sports-related eye injuries, 12.6% of them were using protective equipment for their eyes. Such a low use rate reflected the lack of eye protection awareness among the athletes. At this point, it became very important for coaches to supervise and encourage athletes to use eye-protective devices whenever possible. For instance, eye-protective equipment is usually used in fencing, swimming, and cycling, but it should also be used in other sports.

The present study had limitations. Athletes who participated in the investigation were from Tianjin, which might not reflect the incidence of sports-related eye injuries across China. The numbers of athletes in each sport were different, but it reflected the differences in popularity of different sports. Our study might have missed the most severe injuries because those athletes might have stopped practicing their sport, which might underestimate the true incidence of eye injuries among athletes.

In conclusion, sports-related eye injuries commonly occurred in athletes. Improving the awareness of eye protection, paying more attention to underage athletes, providing financial assistance to athletes from poor families, and avoiding athletes from overwork should be helpful in lowering the incidence of sports-related eye injuries. In the future, stakeholders should pay more attention to sports-related eye injuries in China and propose preventive strategies.

## Data Availability Statement

The raw data supporting the conclusions of this article will be made available by the authors, without undue reservation.

## Ethics Statement

The studies involving human participants were reviewed and approved by the ethics committee of Tianjin Medical University General Hospital. Written informed consent to participate in this study was provided by the participants' legal guardian/next of kin.

## Author Contributions

HY: research design and manuscript preparation. JZ and XZhu: data acquisition, research design, data analysis, and manuscript preparation. ZhiS, JW, and ZhuS: data acquisition, data analysis, and manuscript preparation. JL, TW, YH, RX, HH, XZha, YS, MG, TY, HZ, KH, YLi, YLe, YZ, BC, and YM: data acquisition and manuscript preparation. BH: research design and manuscript preparation. RB: research design and manuscript preparation. All authors approved the final version of this manuscript.

## Funding

This research was supported by National Natural Science Foundation of China (Grant Numbers 82020108007, 81830026) and Beijing-Tianjin-Hebei Special Project (Grant Number 19JCZDJC64300(Z), 20JCZXJC00180).

## Conflict of Interest

The authors declare that the research was conducted in the absence of any commercial or financial relationships that could be construed as a potential conflict of interest.

## Publisher's Note

All claims expressed in this article are solely those of the authors and do not necessarily represent those of their affiliated organizations, or those of the publisher, the editors and the reviewers. Any product that may be evaluated in this article, or claim that may be made by its manufacturer, is not guaranteed or endorsed by the publisher.
